# Wild jackdaws recognise the contact calls of their mate

**DOI:** 10.1007/s10071-025-02020-6

**Published:** 2025-11-26

**Authors:** Victoria E. Lee, Guillam E. McIvor, Alex Thornton

**Affiliations:** 1 Centre for Ecology and Conservation, University of Exeter, Penryn Campus, Penryn, Cornwall, TR10 9FE UK; 2 Department of Behaviour and Cognition, University of Vienna, Vienna, 1030 Austria

**Keywords:** Cognition, Communication, Pair bonding, Recognition, Contact call, Corvid

## Abstract

In stable social environments, the ability to recognise other group members and integrate individual cues with previous experience is likely to be beneficial in mediating social interactions. Under these conditions, selection for individual discrimination and learning of conspecific vocalisations is expected. Recognition may be particularly important in structured societies in which individuals form enduring pair bonds and coordinate biparental care. Many corvids, famed for their sophisticated cognitive abilities, form long-term pair bonds and are considered ideal subjects for investigating the cognitive mechanisms associated with pair bonding; however, evidence that corvids individually recognise their partner under natural conditions remains limited. Here, we tested whether wild jackdaws (*Corvus monedula*), a colonial corvid, discriminate between the contact calls of individual conspecifics. Incubating females were presented with contact call playbacks from their long-term breeding partner, a male from a neighbouring nest, and an unfamiliar male. Females were quicker to respond to the calls of their partner, providing evidence of mate recognition in corvids in the wild.

**Supplementary Information:**

The online version contains supplementary material available at 10.1007/s10071-025-02020-6.

## Introduction

In many animal societies, the ability to recognise individuals is crucial for navigating a changing social world (Tibbetts and Dale 2007; Gokcekus et al. 2021). For example, recognising individual social companions allows group members to avoid conflict by integrating past experience into their current behavioural decision-making (Yorzinski 2017). Recognition is also a prerequisite for tracking the relationships of others and maintaining valuable social bonds (Emery et al. 2007). The latter may be particularly important for species that cooperate with a long-term partner to raise offspring (Dunbar and Shultz 2007; Emery et al. 2007). On the other hand, the socio-cognitive abilities that are hypothesised to be beneficial for pair bonding may carry costs, such as information processing costs (Dall et al. 2005). There is currently limited empirical evidence that identifies the specific costs and benefits associated with these cognitive processes, and how these trade-offs manifest within long-term pair bonds (Scheiber et al. 2008; Sayol et al. 2016; Hooper et al. 2022; Hahn et al. 2025).

From insects to mammals, many animals appear to recognise their social companions, but the mechanisms by which this apparent recognition occurs may vary between species (Tibbetts and Dale 2007). Responses to companions may involve ‘true’ individual recognition - whereby a receiver integrates unique cues from a signaller with information about identity (Tibbetts and Dale 2007; Wiley 2013) – or less specific discrimination based on category-level information about the signaller (Mendelson et al. 2016). Unlike category-level discrimination, where signallers are categorised at a group level (e.g. familiar versus unfamiliar, kin versus non-kin), true individual recognition involves memory of specific, known individuals, allowing recognition of, for instance, a single offspring or a single breeding partner (Tibbetts et al. 2008; Yorzinski 2017; but see Wiley 2013). Although category-level discrimination is widespread across taxa (Tibbetts and Dale 2007), true individual recognition abilities have thus far only been explicitly demonstrated in a small number of mammals (Adachi et al. 2007; Proops et al. 2009; Sliwa et al. 2011; Proops and McComb 2012; Townsend et al. 2012; Sharpe et al. 2013; Kulahci et al. 2014; Gilfillan et al. 2016) and birds (e.g. Wanker and Jennerjahn 1998; Berg et al. 2011; Kondo et al. 2012; Warrington et al. 2015; Nichols and Yorzinski 2016; Colombelli-Négrel and Evans 2017; Farrow et al. 2021; see Yorzinski 2017 for a review). In either case, both true individual recognition and category-level discrimination are expected to facilitate social living by allowing individuals to predict the behaviour of others and adjust their own behaviour accordingly (Yorzinski 2017).

For discrimination to occur, cues must contain a signature that is either unique to the individual (in the case of true individual recognition) and/or the category to which the individual belongs (for category-level discrimination; Yorzinski 2017). Indeed, studies of mammals (McComb and Semple 2005; Pollard and Blumstein 2011; Gustison et al. 2012) and birds (Kroodsma 1977; Freeberg 2006) support the hypothesis that living in more “complex” societies (whether defined by group size, mating system or social structure) is associated with more complex communication systems, in terms of signal repertoire size and/or the amount of social information encoded in these signals (Sewall 2015). With regards to acoustic cues, vocal individuality allows callers to signal their identity and allows receivers to attend to the calls of more reliable individuals. On the other hand, vocal individuality may also carry costs, by allowing receivers or eavesdroppers to target the caller for social punishment and territory disputes (Tibbetts and Dale 2007). As a result, vocal signatures and acoustic recognition abilities are expected to evolve in environments where identity signalling is useful (Tibbetts and Dale 2007; Wiley 2013; Gokcekus et al. 2021). Even in species exhibiting vocal individuality, the nature and extent of this individuality varies: in some cases, vocal individuality is seen in some call types but not others (Rendall et al. 1998; Charrier et al. 2001a); in other cases, the distinguishing features of calls may differ between different call types. For example, the alarm and contact barks of chacma baboons (*Papio ursinus*) both show individual characteristics, but patterns of variation are not consistent between call types (Fischer et al. 2001a).

One type of vocalisation, the contact call, is widely used to advertise identity, coordinate behaviour and maintain group cohesion (Kondo and Watanabe 2009). In many species, contact calls play a vital role in regulating social organisation: as a result, individual recognition of contact calls may be particularly valuable. Contact calls often encode information about individual identity, and many birds and mammals identify social companions (such as mates, Wanker and Jennerjahn 1998; Berg et al. 2011; Guggenberger et al. 2022; kin, Wanker and Jennerjahn 1998; Charrier, Mathevon, et al. 2001b; or individuals, Sharpe et al. 2013) based on contact calls. In many cases, category-level discrimination may be sufficient to identify social companions (e.g. discriminating siblings from other familiar individuals, Wanker and Jennerjahn 1998). However, in some cases, where there are numerous familiar conspecifics within a broad category, more specific recognition mechanisms may be necessary to respond appropriately to particular individuals (e.g. a single mate or parent’s contact calls within a breeding colony: Wanker and Jennerjahn 1998; Charrier et al. 2001b; Berg et al. 2011; Guggenberger et al. 2022). Contact call discrimination of bond partners and kin may be particularly valuable for colonial species (Charrier et al. 2001b; Clark et al. 2006; Guggenberger et al. 2022) and those that engage in biparental care (Emery et al. 2007), while contact call discrimination beyond immediate pair or kin bonds allows individuals to navigate relationships that change over time (Kondo and Watanabe 2009; Sewall 2015).

In this study, we investigated contact call discrimination in wild jackdaws (*Corvus monedula*), a highly social corvid. Famed for their sophisticated cognitive abilities (Emery and Clayton 2004; Güntürkün and Bugnyar 2016), corvids provide an ideal system in which to test the cognitive demands associated with social life (Emery et al. 2007). Many corvids form long-term pair bonds, with pairs working together to acquire resources and raise young (Röell 1978; Emery et al. 2007; Clayton and Emery 2007; Bugnyar 2013; Jolles et al. 2013b; Hahn et al. 2025): mate recognition is therefore likely to be a vital prerequisite for many of these activities. Several species, including jackdaws, exhibit fission-fusion social dynamics where advertising identity is predicted to be most valuable (Kondo and Watanabe 2009), and where recognition within and beyond the pair bond is likely to be beneficial e.g. in large flocks (Jolles et al. 2013a; Ling et al. 2019), dominance hierarchies (Kondo and Watanabe 2009; but see Wiley 2013) and dynamic social decision-making (Kings et al. 2023). In support of this, acoustic analyses and playback experiments demonstrate that the calls of many corvid species encode information about individual identity, and that individuals discriminate between conspecific calls (American crow *Corvus brachyrhynchos*, Mates et al. 2015; pinyon jay *Gymnorhinus cyanocephalus*, McArthur 1982; rook *Corvus frugilegus*, Røskaft and Espmark 1984; large-billed crow *Corvus macrorhynchos*, Kondo et al. 2012; Mexican jay *Aphelocoma ultramarina*, Hopp et al. 2001). Previous studies have also shown that the food calls (Zandberg et al. 2014) and alarm calls (Woods et al. 2018) of jackdaws are individually distinct, and that birds respond preferentially to the antipredator recruitment calls of colony members over the calls of unfamiliar individuals (Woods et al. 2018). Alongside these more context-specific calls, contact calls are used widely by adult jackdaws. Contact calls are heard frequently in jackdaw breeding colonies and social foraging groups, and males will often make contact calls when returning to the nest to provision incubating females. Because these calls are used in a range of social contexts, contact calls are a likely cue for advertising identity and mediating social interactions. Given the importance of pair coordination for jackdaws (e.g. during offspring provisioning, Henderson et al. 2000; foraging, Kings et al. 2023; nest building, Hahn et al. 2021; and collective movement, Ling et al. 2019), contact calls are likely to be used in mate recognition. Furthermore, this mate recognition may involve recognition based on the memory of a specific partner: due to the abundance of other familiar birds in the breeding colony, category-level discrimination may not be sufficient for accurate mate recognition in this context. However, the function of jackdaw contact calls has not been experimentally tested: acoustic analyses have identified structural differences in the contact calls of individuals (Stowell et al. 2016), but it is unknown whether jackdaws perceive these differences, or how they respond to the contact calls of different conspecifics. Behavioural experiments of corvids to date have focused on discrimination of familiar individuals (Hopp et al. 2001; Kondo et al. 2012; Zandberg et al. 2014; Woods et al. 2018; Coomes et al. 2019), offspring (McArthur 1982) or siblings (Røskaft and Espmark 1984); as such, no studies have explicitly tested recognition within the corvid pair bond, or the cues used to do so. Furthermore, although captive large-billed crows (*Corvus macrorhynchos*) have been shown to individually recognise familiar conspecifics (Kondo et al. 2012), individual recognition among corvids has yet to be tested under natural conditions. As captive populations are often small, with individuals housed in close proximity for extended periods of time, it is possible that subjects may have more opportunity to learn each other’s vocalisations; field studies are therefore needed to complement findings from laboratory systems. To address these research gaps, we used playback experiments to investigate the response of free-living female jackdaws to the contact calls of (i) their male partner; (ii) a familiar male from a neighbouring nest; (iii) an unfamiliar male. We predicted that if females discriminate between individuals based on contact calls, subjects would vary in their behavioural response to playbacks depending on the identity of the caller. Specifically, we predicted that females would respond more quickly to the contact call of their partner signalling the arrival of food, and show longer response latencies for unfamiliar contact calls than for those of their partner and neighbour (lower response latencies reflect more efficient processing of familiar stimuli, see Miller et al. 2005; Landi and Freiwald 2017; Ramon and Gobbini 2018). We also predicted that following the initial response, females may show a stronger overall response to the contact calls of unfamiliar individuals (e.g. by leaving the nest box to obtain more information about the potential intruder).

## Methods

### Study population

This experiment was conducted during the breeding season (2015–2017) using free-living, individually colour-ringed jackdaws nesting in boxes provided by the Cornish Jackdaw Project (University of Exeter, Cornwall, UK). The Cornish Jackdaw Project comprises three main study sites: Stithians (a village churchyard; 50°11′26″N, 5°10′51″W; 33 nest boxes), Pencoose Farm (50°11′56″N, 5°10′9″W; 35 nest boxes) and the University of Exeter’s Penryn campus (50⁰17’32”N; 5⁰11’96”W; 11 nest boxes). Due to the close proximity of the Stithians & Pencoose sites (1.5 km), some population crossover does occur with birds nesting at one site observed foraging at the other. For the purposes of this study it was assumed, based on extensive observations (of > 4000 ringed individuals over 12 years), that breeding birds from the Campus site (5 km) had no contact with birds from Pencoose and Stithians as the populations are known to roost in separate locations (pers. obs., unpublished data). In other populations, jackdaws have also shown reasonably strong site fidelity once they start to breed (Röell 1978).

### Playback experiments

#### Audio recordings

Early in the nest-building phase (late March - early April), nest boxes occupied by breeding jackdaws were fitted with CCTV cameras and lapel microphones (AKG-C417PP) concealed behind a panel. For playback experiments, focal nest boxes were selected with at least one colour-ringed individual (to ensure accurate identification of vocalising birds), and close neighbours (to ensure that the neighbouring male’s contact call would be an ecologically relevant stimulus for the focal female). Audio recordings were made early in the morning (start time: 0700–0900) during late March and early April, when birds were engaged in nest building or early egg incubation. Video recordings (using JXD 990 digital video recorders) and audio recordings (using Olympus LS-100 & Tascam DR-100MKII PCM recorders) were taken daily as required. Each recording ran for around 3.5 h, when researcher activity at study sites was at a minimum. Where available, contact call exemplars were also extracted from recordings obtained using an identical protocol during previous seasons (2013–2015).

#### Call extraction

Audio recordings and extensive observations show that both males and females call frequently during their regular visits to the nest box. Clear, high-quality exemplars of male contact calls with minimal background noise were extracted from nest box audio recordings and normalised for amplitude using Audacity (audacity.sourceforge.net). Extracted calls were arranged into playback files comprising two bouts of three contact calls: within-bout, calls occurred at 2 s intervals to simulate natural calling, with a 10 s pause between the two call bouts to maximise the opportunities for females to derive information from males’ contact calls in the field (supplementary figure S1). Where possible, playback files contained six different contact calls (range: 2–6 calls) from each male, with preference given to calls recorded on different days. Where fewer than six male contact calls were available, the number of repeated calls was kept as low as possible and call sequences were modified to ensure that focal females would not hear repeated calls presented in the same order.

Each focal female was assigned three playback files: one containing the contact calls of her partner (‘Partner’ treatment), a second containing contact calls of a male from a neighbouring nest box < 50 m away (‘Neighbour’ treatment) and a third containing contact calls from an unfamiliar male at a different colony (‘Stranger’ treatment). Because of their close geographical proximity and the observed movement of birds between Stithians and Pencoose, only contact calls obtained from the Campus were used in the ‘Stranger’ treatments at these sites.

#### Experimental trials

Early in the breeding season, a ‘decoy’ loudspeaker (plastic bottle wrapped in vegetation) was mounted on a hook adjacent to each focal nest box, to encourage habituation and minimise any neophobic behaviour in response to the loudspeaker itself. On the day of the experiment, decoys were replaced with a remote-controlled loudspeaker (FoxPro Fury 2) wrapped in similar vegetation, using a decorators’ pole to minimise disturbance at the nest box. Video recording equipment was also set up (DVR JXD 990) to record female behaviour inside the nest box. Following setup, the experimenter returned to a concealed location at least 50 m away to control the loudspeaker and make additional behavioural observations. To allow birds to return to normal behaviour, a baseline period of at least 20 min elapsed between the female’s first return to the nest box and presentation of the first playback stimulus. Playbacks only occurred when the focal female was inside the nest box for at least 5 min with no disturbance (including male visits). The order in which focal females received each playback treatment was counterbalanced across the experiment.

### Video coding

Video coding was carried out in BORIS (Friard and Gamba 2016). We recorded the frequency and duration of all behaviours exhibited by the focal female in the two-minute period following each playback presentation. These included looking at the nest box entrance, arranging nest material, peeking out of the nest box entrance and leaving the nest box. All playbacks occurred at least 5 min after the last visit by the male, and no males visited the nest box in the two minutes following playback. In total, we carried out 57 trials at 19 focal nest boxes; two trials were subsequently discounted (one due to camera failure, and one trial where the focal female appeared to be asleep during the playback presentation).

20% of videos were analysed by a second coder who was blind to treatment. Inter-rater reliability was analysed using the *irr* package in R (Gamer et al. 2012), and coders showed a high degree of agreement for all behaviours analysed (latency to look at the nest box entrance following playback: ICC = 0.95, *p* < 0.001; time spent looking at and peeking out of the nest box entrance in the two minutes following playback: ICC = 0.92; *p* < 0.001. In 11/12 cases, coders agreed on the extent of female response to the playback; see ‘Behavioural response to playback’).

### Statistical analyses

All analyses were carried out in R (R Core Team 2017), with models built using the *lme4* (Bates et al. 2015) and *ordinal* (Christensen 2018) packages. Models were simplified using log-likelihood ratio tests, following examination of model plots to ensure assumptions (homogeneity and normality of residuals) were met. Unless otherwise stated, sample sizes for models are comprised of 55 observations from 19 females (see *Video coding*).

#### Latency to look at nest box entrance

In studies of recognition, the latency of test subjects to respond to stimuli is considered to reflect information processing time, as familiar cues (e.g. faces) are processed more efficiently than unfamiliar stimuli (Miller et al. 2005; Landi and Freiwald 2017; Ramon and Gobbini 2018). Here, we expected females to respond rapidly to the contact calls of their own mate, especially as this signals the arrival of food; due to the increased processing time associated with unfamiliar contact calls, we also predicted that females would exhibit the longest response latencies in the ‘Stranger’ treatment.

In most cases, females initially responded to playbacks by looking towards the nest box entrance (69%). Female tendency to look towards the nest box entrance (1 = yes, 0 = no) was analysed using a binomial mixed model, with treatment (Partner, Neighbour or Stranger) and trial number (1–3) included as fixed effects and female ID as a random term. A similar model investigated the latency of females to look towards the nest box entrance (for those that did so), using a Gaussian error distribution, following log-transformation of the response variable. This model included 42 trials from 19 females, after excluding those that did not look towards the nest box entrance within one minute of the playback.

#### Behavioural response to playback

Although most females initially responded to playbacks by looking at the nest box entrance (LOOK), some individuals subsequently went on to peek out of the nest box entrance (PEEK) or leave the nest box (EXIT). The extent of female response was analysed using a cumulative link mixed model (CLMM) with an ordinal response term (NONE/LOOK/PEEK/EXIT). Treatment (Partner, Neighbour, Stranger) and trial number (1–3) were included as fixed effects, and female ID as a random term. To investigate whether individual identity influenced female response, the mixed model was compared to a cumulative link model (CLM) without the random term (Christensen 2015).

#### Time spent looking and peeking following playback

The time that females spent looking at or peeking out of the nest box entrance in the two minutes following the start of the playback presentation was analysed using an LMM with a Gaussian error distribution (following box-cox transformation of the response variable). Treatment (Partner, Neighbour, Stranger) and trial number (1–3) were included as fixed effects, and female ID as a random term. This model comprised 54 observations from 19 females (an additional trial was dropped from the analysis as the focal female immediately left the nest box upon hearing the playback).

## Results

### Latency to look at nest box entrance

In 13 cases, the female did not look towards the nest box entrance within one minute of the playback. The probability of a female looking towards the nest box entrance did not differ significantly between treatments (X^2^ = 2.72, df = 2, *p* = 0.299) and was not influenced by playback order (X^2^ < 0.01, df = 2, *p* = 0.964; Table S1).

**Fig. 1:**
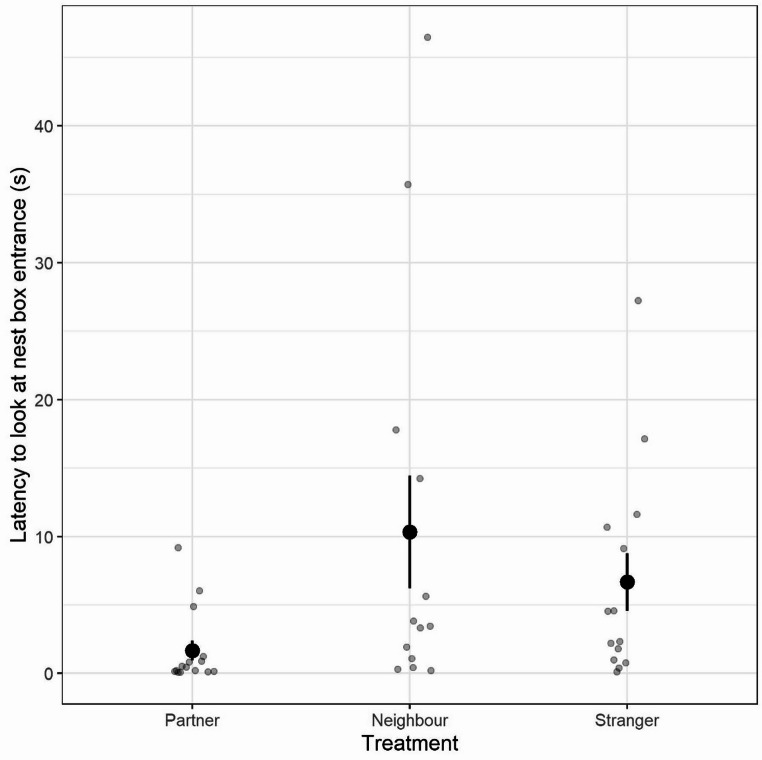
Latency of focal females to look towards the nest box entrance following playbacks, by treatment group. Grey circles denote individual data points, points and whiskers denote group means and standard error

For the females that did look towards the nest box entrance (*n* = 42), the identity of the calling male influenced their latency to do so (X^2^ = 11.50, df = 2, *p* = 0.003; Fig. 1; Table 1). Females were substantially quicker to look towards the entrance following playback of their partner’s contact calls (mean ± SE: 1.7 ± 0.7s), responding more quickly than to contact call playbacks of neighbouring males and unfamiliar males (mean ± SE: neighbour treatment = 10.3 ± 4.1s; stranger treatment = 6.7 ± 2.1s).

**Table 1:** Output of GLMM investigating the effect of treatment and trial number on the latency of females to look towards the nest box entrance after playback. Values are derived from full model with significant effects shown in italics (*n* = 42 observations from 19 females)

Fixed effects		β	SE	t-value
Intercept		−0.27	0.75	−0.36
*Treatment*	*Partner (reference)*			
	*Neighbour*	*1.85*	*0.60*	*3.07*
	*Stranger*	*1.78*	*0.59*	*3.05*
Trial number		−0.21	0.30	−0.72
**Random effects**			**Variance**	**SD**
Female ID			< 0.001	< 0.001
Residual			2.48	1.57

### Behavioural response to playback

**Fig. 2:**
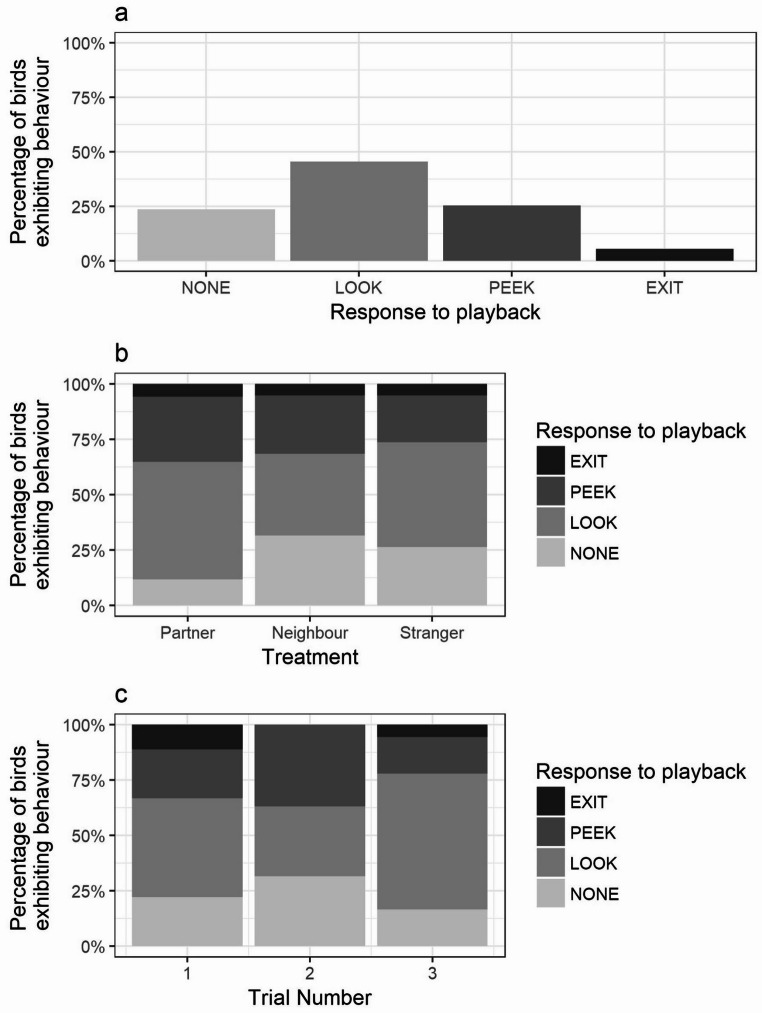
Percentage of females exhibiting each type of behavioural response to playbacks (LOOK: looking out of nest box entrance; PEEK: peeking out of nest box entrance; EXIT: leaving the nest box). Panels show proportion of females exhibiting each behavioural response (**a**) across all trials; (**b**) by treatment group (contact call playbacks from partner, neighbour or unfamiliar male); (**c**) by trial number (1–3)

Females responded to playbacks by looking at the nest box entrance (LOOK), peeking out of the nest box entrance from a standing position (PEEK) and/or leaving the nest box (EXIT) (Fig. 2a). The extent of the response shown by the female (NO RESPONSE < LOOK < PEEK < EXIT) was not influenced by trial number (X^2^ = 0.07; df = 1; *p* = 0.79; Fig. 2c; Table 2) or the identity of the caller in the playback (X^2^ = 2.04; df = 2; *p* = 0.36; Fig. 2b; Table 2). The identity of the focal female did not significantly predict subjects’ behavioural response to playbacks (X^2^ = 1.44; df = 1; *p* = 0.23; Table 2). Leaving the nest box was a rare response to the playback, occurring once in each treatment (Fig. 2b). One female left the nest box following playback of a stranger’s contact calls, while another female left following playback of their partner’s and neighbour’s contact calls (and peeked out of the nest box entrance in response to a stranger’s contact calls).

**Table 2:** Output of CLMM investigating the effect of treatment group and trial number on the behavioural responses of focal females to playbacks. Values are derived from full model (*n* = 55 observations from 19 females)

Model parameters	β	SE	z-value	*p*-value
*Threshold (response)*				
NONE|LOOK	−2.33	0.99	−2.35	0.02
LOOK|PEEK	0.09	0.90	0.09	0.92
PEEK|EXIT	2.49	1.07	2.33	0.02
*Treatment*				
Partner (reference)	−0.81	0.68	−1.19	0.23
Neighbour	−0.85	0.67	−1.27	0.20
Stranger	−0.15	0.33	−0.44	0.66
*Trial number*	−0.81	0.68	−1.19	0.23
**Random effects**			**Variance**	**SD**
Female ID			1.03	1.01

### Time spent looking/peeking following playback

**Fig. 3:**
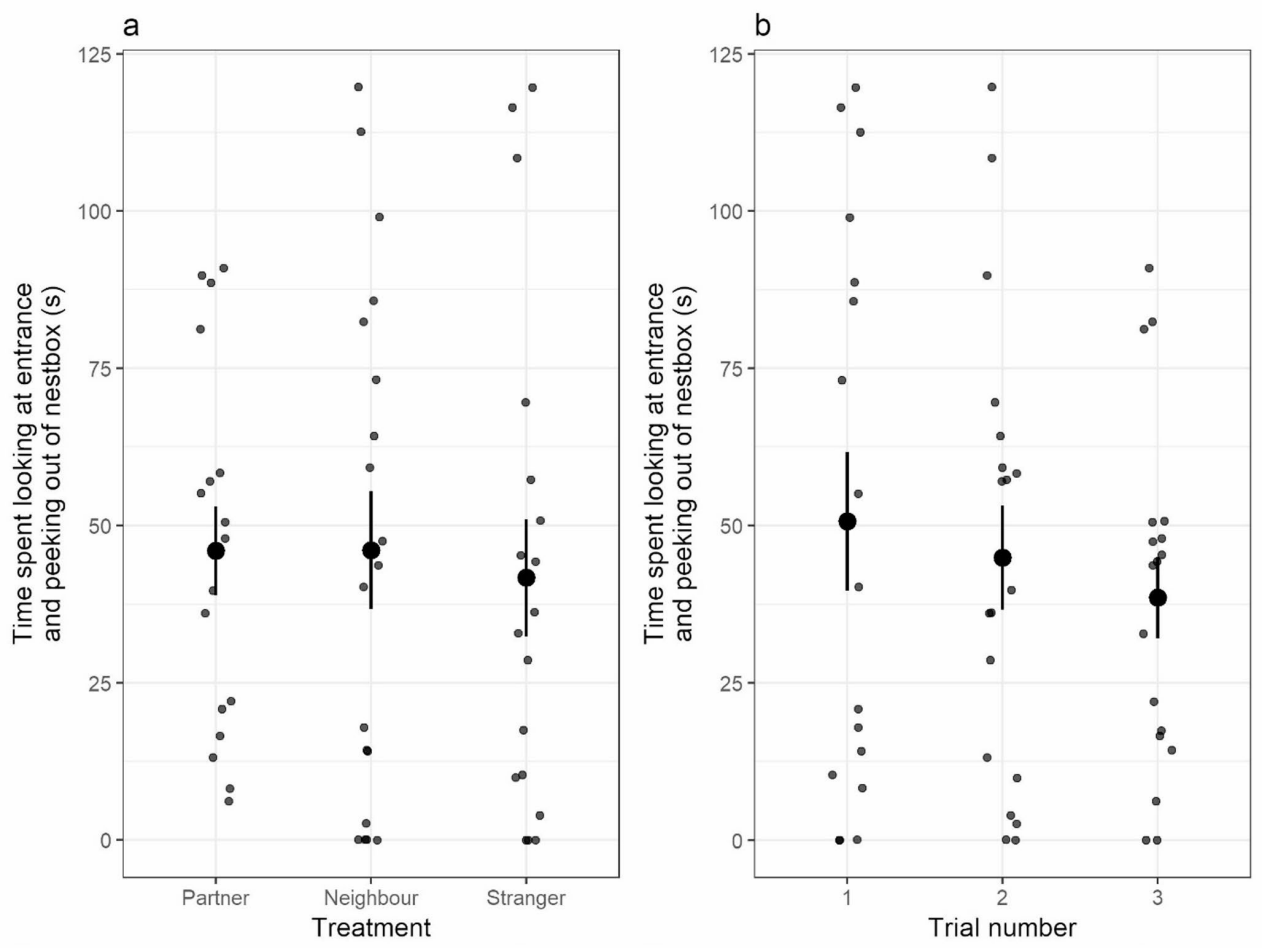
Time spent by females looking at or out of the nest box entrance in the two minutes following playbacks, by (**a**) treatment and (**b**) trial number. Grey circles show individual data points, black points and whiskers denote mean and standard error

The time females spent looking at or out of the nest box entrance in the two minutes following playbacks was broadly similar across trials (X^2^ = 0.12; df = 1; *p* = 0.73; Fig. 3b; Table 3) and was not significantly influenced by the identity of the caller (X^2^ = 1.40; df = 2; *p* = 0.50; Fig. 3a; Table 3).

**Table 3:** Output of GLMM investigating the effect of treatment and trial number on the time spent by females looking at or out of the nest box entrance in the two minutes following playback presentations. Values derived from full model (*n* = 54 observations from 19 females)

Fixed effects		β	SE	t-value
Intercept		9.88	2.23	4.44
Treatment	Partner (reference)			
	Neighbour	−1.56	1.64	−0.95
	Stranger	−1.71	1.64	−1.04
Trial number		−0.39	0.84	−0.47
**Random effects**			**Variance**	**SD**
Female ID			4.77	2.18
Residual			23.07	4.80

## Discussion

This experiment aimed to determine whether female jackdaws recognise the contact calls of their bonded partner, and discriminate between the contact calls of familiar and unfamiliar males. In line with predictions, female jackdaws differed in their response to playbacks depending on the identity of the caller. Incubating females were much quicker to look towards the entrance of their nest box upon hearing the contact calls of their partner compared to those of other males, likely anticipating the arrival of food. Upon hearing playbacks, although females initially responded by looking towards the nest box entrance, many females went on to peek out of the nest box or leave the nest box. However, the extent of female response was not influenced by the identity of the caller. The identity of the caller also had no significant effect on the length of time females spent looking at or out of the nest box entrance after hearing the playback. These findings demonstrate that female jackdaws individually recognise the contact call of their single, long-term breeding partner; providing experimental evidence of partner recognition among corvids in the wild (Tibbetts et al. 2008; Yorzinski 2017).

Recognition of a partner’s contact call is likely to be beneficial in the social life of jackdaws. In this species, pair bonds persist for many years, with individuals coordinating behaviour to raise young and defend a nest site. Biparental care is vital for reproductive success, with the loss of a partner during offspring provisioning often resulting in brood failure (Röell 1978; Henderson et al. 2000). As a colonial species, jackdaws may also derive benefits from recognising their partner in a crowd (Aubin and Jouventin 1998; Kondo and Watanabe 2009). Adult breeding pairs often forage together (Valletta et al. 2017) and join large mixed-species winter flocks of hundreds, or even thousands of individuals. Analyses of flocking dynamics suggest that individuals travel close to their partner within these flocks (Jolles et al. 2013a; Ling et al. 2019). Given that contact calls are frequently given in flight, this provides a potential mechanism by which individuals might maintain pair cohesion even when flying at high speed among hundreds of other birds. Similarly, experiments using computerised feeders show that jackdaws strategically adjust their social relationships to access the best resources during social foraging, but continue to visit feeders with their partner regardless of reward outcomes (Kings et al. 2023). This implies that jackdaws must recognise one another as individuals: given that contact calls are frequently used by jackdaws in a range of contexts, they are likely to play an important role in coordinating joint foraging by pair-bonded partners. The results of this study suggest that incubating females respond to the contact calls of their partner in a context which often signals the arrival of food (although jackdaws also use an additional type of call that is specific to food-sharing). Conducting similar playback experiments in other social situations may shed light on the wider function of these calls, and the extent to which partner recognition mechanisms operate in different contexts.

In many species, conspecific recognition occurs via relatively simple mechanisms, such as category-level discrimination (see Wiley 2013). However, more complex forms of ‘true’ individual recognition are thought to be relatively cognitively demanding (Tibbetts and Dale 2007; Yorzinski 2017). Although true individual recognition has been demonstrated in several mammals and birds (reviewed in Yorzinski 2017), only one other study to date has explicitly investigated these abilities experimentally in corvids. Violation-of-expectation experiments by Kondo et al. (2012) demonstrate that captive large-billed crows (*Corvus macrorhynchos*) recognise group members individually, responding more strongly when a visual presentation of a social companion is combined with the ‘incorrect’ vocalisation. In our study, females responded more quickly to the calls of their partner than to those of a neighbouring male or a stranger. As male jackdaws are responsible for provisioning females during incubation, this suggests that females recognise their breeding partner’s call, likely anticipating the imminent arrival of food. Given that females hear the calls of males from neighbouring nest boxes many times a day, the difference in the latency to respond to mates versus neighbours is unlikely to be driven by differences in familiarity. Rather, our results imply that jackdaws may show ‘true’ individual recognition to identify their partner under natural conditions, though further study – for instance using violation-of-expectation paradigms - is needed for definitive confirmation (cf. Guggenberger et al. 2022).

It is surprising that test subjects did not appear to differ greatly in their response to the contact calls of neighbours and unfamiliar males. Whilst this could be interpreted as an inability to discriminate contact calls beyond those of an immediate partner, it may also be that there is no specific need for females to respond differently to the contact calls of neighbours and strangers. The fact that jackdaws have previously been shown to discriminate between familiar and unfamiliar alarm calls (Woods et al. 2018) suggests that the latter explanation is more likely. For example, if unfamiliar individuals are encountered frequently, if neighbours and strangers are unlikely to pose a threat, or if contact calls are associated with a lack of threat (Lee et al. 2019), then their calls may not elicit different responses from the incubating female. Lack of motivation may also explain why playbacks of partner contact calls did not influence females’ responses beyond initially looking towards the nest box entrance: males frequently return to the nest box during the incubation stage, and these visits may not require the female to gather additional information. The failure of test subjects to respond as predicted in studies of vocal recognition may not necessarily reflect an inability to discriminate between the calls of different individuals, but rather that distinguishing between distinct vocalisations may not be relevant in a given context (Fischer et al. 2001b; Townsend et al. 2010; Jansen et al. 2013). For example, meerkats (*Suricata suricatta*) do not appear to attend to individual signatures encoded in alarm calls, possibly as these calls are a highly reliable signal of an immediate mortal threat (Schibler and Manser 2007). Taken together, these findings highlight the need for behavioural experiments to complement analyses of individuality in call structure. Further work investigating the acoustic individuality of contact call structure in this species would also be valuable, and may further support the behavioural findings presented here.

The idea that monogamous pair bonds may act as a potential driver for cognitive evolution has received widespread attention, but still lacks clear empirical support (Scheiber et al. 2008; Hooper et al. 2022; Hahn et al. 2025). Although corvids are considered likely candidates for testing these theories (Emery et al. 2007), the basic assumption that individuals recognise their bonded partner has not been widely tested in natural populations (Colombelli-Négrel and Evans 2017; Guggenberger et al. 2022). Research suggests that corvid pair bonds are highly valuable relationships, with individuals investing significant time and energy in affiliative behaviours and agonistic support (Röell 1978; Emery et al. 2007; Fraser and Bugnyar 2012; Kubitza et al. 2015; Boucherie et al. 2018; but see Hooper et al. 2021), although our understanding of the fitness consequences is limited. If maintaining a pair bond is cognitively demanding but yields important fitness benefits, this may shed light on the evolution of corvids’ sophisticated cognitive abilities (Emery et al. 2007). However, the theory of ‘relationship intelligence’ remains speculative, as precisely whether and how maintaining pair bonds is cognitively demanding is seldom tested empirically (Hahn et al. 2025). The cognitive demands of conspecific recognition provide a good starting point: for example, does relationship intelligence require explicit recognition abilities, or are simpler discrimination mechanisms sufficient (Mendelson et al. 2016)? Several methodological approaches can be used to investigate individual recognition abilities, including experiments involving presentation of multi-modal stimuli (Yorzinski 2017). Due to the logistical challenges associated with implementing these experiments in the field, studies of captive individuals under controlled conditions are particularly valuable in this regard. On the other hand, captive populations may not always provide an accurate representation of a species’ cognitive performance: small group sizes and physical proximity may allow individuals to become more familiar with each other’s vocalisations. Consequently, captive studies must be complemented by field experiments where multiple cues compete for subjects’ attention, and findings are more reflective of natural behaviour (Thornton and Truskanov 2022). Finally, although corvids and parrots provide ideal opportunities to test relationship intelligence (Emery et al. 2007), research should not be limited to these systems. By broadening our focus to other species with long-term pair bonds (such as geese; Guggenberger et al. 2022), we may be able to determine whether the nature of these pair bonds, and the cognitive demands associated with them, truly differ from those of supposedly more ‘cognitively advanced’ species (Scheiber et al. 2008; Thornton 2014).

## Supplementary Information

Below is the link to the electronic supplementary material.

Supplementary Material 1

## Data Availability

The data associated with this study are available in the Figshare repository: https:/figshare.com/s/95043dec47e4152d66b2.
