# Supplementary material for: Wild jackdaws recognise the contact calls of their mate
Source: Anim Cogn. 2025 Nov 26;28(1):97. doi: 10.1007/s10071-025-02020-6 (PMC12657572; doi:10.1007/s10071-025-02020-6)
Supplement: Supplementary file 1 — Supplementary Material 1 [file 10071_2025_2020_MOESM1_ESM.docx]

**Supplementary material: Wild jackdaws recognise the contact calls of their mate**

Victoria E. Lee^a^, Guillam E. McIvor^a,b^ & Alex Thornton^a^*

^a^Centre for Ecology and Conservation, University of Exeter, Penryn Campus, Penryn, Cornwall, UK TR10 9FE.

^b^Department of Behaviour and Cognition, University of Vienna, 1030 Vienna, Austria.

*Email correspondence: [victoria.lee2212@gmail.com](mailto:victoria.lee2212@gmail.com) / [alex.thornton@exeter.ac.uk](mailto:alex.thornton@exeter.ac.uk)

**
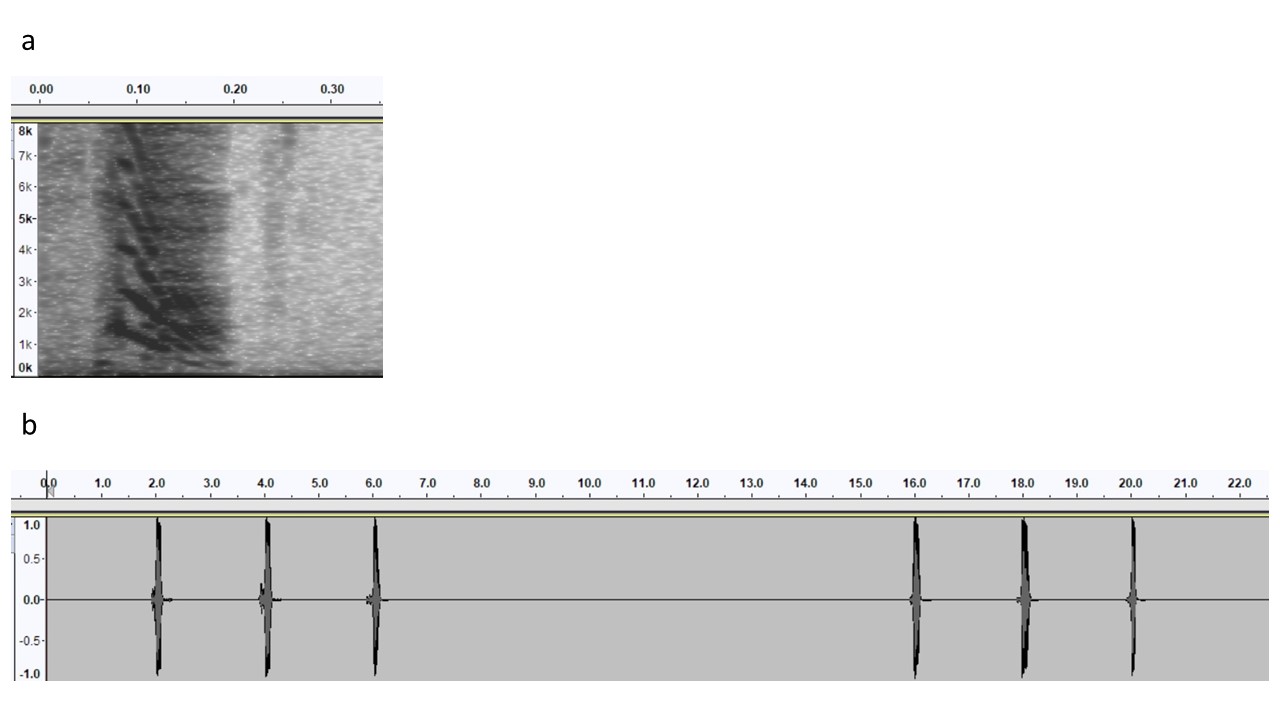
**

**Figure S1:** (a) Spectrogram of a contact call from a male jackdaw (b) waveform of a playback track, comprised of six contact calls from a male jackdaw.

**Table S1:** Output of GLMM investigating the effect of treatment and trial number on the probability that females would look towards the nest box entrance after playback. Values are derived from full model (n=55 observations from 19 females).

| **Fixed effects** | | **β** | **SE** | **z-value** |
| --- | --- | --- | --- | --- |
| Intercept | | 2.29 | 1.42 | 1.61 |
| Treatment | Partner (reference) |  |  |  |
|  | Neighbour | -1.41 | 1.02 | -1.39 |
|  | Stranger | -1.12 | 0.99 | -1.13 |
| Trial number | | 0.02 | 0.44 | 0.04 |
| **Random effects** | | | **Variance** | **SD** |
| Female ID | | | 0.77 | 0.88 |
|  | | |  |  |
